# Nomogram based on clinical and laboratory characteristics of euploid embryos using the data in PGT-A: a euploid-prediction model

**DOI:** 10.1186/s12884-022-04569-3

**Published:** 2022-03-17

**Authors:** Xitong Liu

**Affiliations:** The Assisted Reproduction Center, Northwest Women’s and Children’s Hospital, Xi’an, China

**Keywords:** Euploid, Pre-implantation genetic testing for aneuploidy (PGT-A), Nomogram, Prediction model

## Abstract

**Background:**

The evaluation of embryo morphology may be inaccurate. A euploid prediction model is needed to provide the best and individualized counseling about embryo selection based on patients and embryo characteristics.

**Methods:**

Our objective was to develop a euploid-prediction model for evaluating blastocyst embryos, based on data from a large cohort of patients undergoing pre-implantation genetic testing for aneuploidy (PGT-A). This retrospective, single-center cohort study included data from 1610 blastocysts which were performed PGT-A with known genetic outcomes. The study population was divided into the training and validation cohorts in a 3:1 ratio. The performance of the euploid-prediction model was quantified using the area under the receiver operating characteristic (ROC) curve (AUC). In addition, a nomogram was drawn to provide quantitative and convenient tools in predicting euploid.

**Results:**

We developed a reliable euploid-prediction model and can directly assess the probability of euploid with the AUC (95%CI) of 0.859 (0.834,0.872) in the training cohort, and 0.852 (0.831,0.879) in the validation cohort, respectively. The euploid-prediction model showed sensitivities of 0.903 and specificities of 0.578.

**Conclusions:**

The euploid-prediction model is a reliable prediction model and can directly assess the probability of euploid.

**Supplementary Information:**

The online version contains supplementary material available at 10.1186/s12884-022-04569-3.

## Background

In vitro fertilization (IVF) has been a common infertility treatment for infertile couples. The selection of embryos for transfer has relied upon standard morphology grading as the first-line method [1]. As elective single embryo transfer (eSET) has been widely advocated, extending embryo culture to the blastocyst stage allows for better evaluation of the implantation potential of the embryo [2]. The high incidence of chromosome aneuploidy in human embryos is a major reason for implantation failure and miscarriage [3]. The prevalence of aneuploidy is greater earlier in gestation than live birth because chromosomal abnormalities account for a large proportion of early miscarriage [4]. However, morphologic assessment cannot accurately ascertain the embryo’s chromosome status.

The application of trophectoderm biopsy and preimplantation genetic testing for aneuploidy (PGT-A) has recently been used worldwide to improve IVF outcomes. Indications for patients undergoing PGT-A include advanced maternal age, recurrent pregnancy loss, or recurrent implantation failure. Trophectoderm biopsy of 5–10 cells is required to obtain embryonic genetic material. One randomized controlled trial (RCT) showed a significant increase in ongoing pregnancy rates and live birth rates with the use of PGT-A than morphology alone [5].

With the use of various techniques, especially next-generation sequencing (NGS), the sensitivity and resolution of copy number variation genome-wide have been increased [6]. However, a major limitation of PGT-A is invasive and time consuming and sophisticated in terms of operation [7]. Recent study showed time-lapse imaging as a non-invasive approach for superior method of embryo selecting, however, this technology is not cheap [8]. Much attention has been focused on improving methods for morphological selection of embryos with high implantation potential.

Prediction models are developed by using the combination of features to help clinical decision-making [9]. However, in the context of euploidy embryo selection, the clinical application of the prediction model in IVF is limited because the algorithms usually do not combine comprehensive maternal and embryonic characteristics. In addition, most studies only evaluate embryos that were transferred, however, the implantation potential of embryos that were not transferred has been eliminated.

Given the previous findings, we hypothesized that morphokinetic parameters could predict euploid embryos. This study combines both clinical and embryonic features for euploid embryo prediction. Our study is based on the data from a large cohort of patients treated with PGT-A at the blastocyst stage. The objective of the study was to develop a nomogram based on clinical and laboratory characteristics that could predict the probability of euploid embryos.

## Materials and methods

### Study design and population

Institutional review board approval of the Northwest women’s and children’s hospital was obtained (number 2021002). Patients undergoing PGT-A from April 2016 to June 2019 at the department of the assisted reproductive center of Northwest Women’s and Children’s Hospital were reviewed for inclusion in the dataset. 409 patients in 398 IVF cycles were included. A total of 1610 blastocyst embryos met all inclusion criteria. Exclusion criteria included patients with no available embryos and oocyte recipients.

### Ovarian stimulation and hCG trigger

The use of the protocol for ovarian stimulation was chosen based on the evaluation of the ovarian reserve tests including antral follicle count (AFC), basal FSH (bFSH), and anti-Mullerian hormone (AMH). The GnRH antagonist and GnRH agonist long protocols have been described elsewhere [10]. Briefly, for the GnRH antagonist protocol, recombinant follicle-stimulating hormone (rFSH) was started at 150 – 225 IU/day as gonadotropin stimulation on day 2 of the menstrual cycle. The dose of rFSH could be adjusted up to a maximal dose of 450 IU/day according to ovarian response. 250 mg of GnRH antagonist was added when the dominant follicle exceeded 12–14 mm. For the GnRH agonist long protocol, patients received 0.05–0.1 mg of GnRH agonist from the mid-luteal phase of the previous cycle until the hCG trigger. After menstrual bleeding, when the pituitary desensitization had reached, gonadotropin stimulation was started as GnRH antagonist protocol. When two or more leading follicles reached 17 mm, hCG triggering for final oocyte maturation was performed. Oocyte retrieval was performed 36 h after the hCG trigger.

### Insemination and embryo culture

Insemination of retrieved oocytes was done by intracytoplasmic sperm injection (ICSI). All the embryos were placed in pre-equilibrated culture dishes (EmbryoSlide, Vitrolife) under oil at 37 °C and 5.5% CO_2_ in air in the time-lapse incubator (EmbryoScope, Vitrolife). Laser-assisted breaching of the zona pellucida was performed on day 3. Embryos were cultured to the blastocyst stage in the standard incubators at 37 °C. Embryos were monitored with the Primo Vision time-lapse system. Imaging frequency was set to 10-min intervals with multiple focal planes recorded every 60 min.

### Trophectoderm biopsy

The embryos were assessed on day 5 and 6, and the trophectoderm (TE) biopsy was performed. Biopsied TE cells were then stored at − 20 °C for future whole genome amplification (WGA) and next-generation sequencing (NGS). After the biopsy, blastocysts were vitrified to be replaced in the subsequent frozen-thawed embryo transfer cycle.

### Embryo scoring

Embryo morphology was assessed by two well-trained embryologists as described elsewhere [11]. Assessment of fertilization is carried out about 16-18 h (day 1) after fertilization. Oocytes are classed as fertilized if two pronuclei (2PN) are present and the second polar body has been extruded. Other oocytes are classified as abnormally fertilized (0PN, 1PN, 3PN). After the evaluation on day 1, zygotes are left in culture for a further 48 h, and the cleavage embryo quality will be observed at 72 (day 3) hours after oocyte retrieval. The embryos are scored according to a combination of blastomere number, blastomere size, and fragmentation. Based on the uniformity of the blastomeres, the embryos without significant differences in blastomere volume were classified as grade I, embryos with one to two significant differences were classified as grade II, and embryos with two or more significant differences were classified as grade III. Based on an embryo’s fragmentation, four grades were classified: grade I, embryos with < 10% fragmentation; grade II, embryos with 10–20% fragmentation; grade III, embryos with 20–30% fragmentation; and grade IV, embryos with > 30% fragmentation. Embryo grades were scored comprehensively according to the above three criteria. Each blastocyst evaluation was performed according to Gardner Grade Standard on day 5 or 6 [12]. Briefly, blastocysts were given a score from 1 to 6 based on the expansion degree and hatching status. 1 = early blastocyst with less than half of the volume of the embryo. 2 = blastocyst with half or greater volume of the embryo. 3 = full blastocyst completely filling the embryo. 4 = expanded blastocyst larger than the early embryo. 5 = hatching blastocyst with the trophectoderm starting to herniate through the zona. 6 = hatched blastocyst. For blastocysts graded as 3–6, the development of the inner cell mass (ICM) was assessed as A, B, and C. A = tightly packed, many cells. B = loosely grouped, several cells. C = very few cells. Trophectoderm (TE) was evaluated as A, B, and C. A = many cells forming a cohesive epithelium. B = few cells forming a loose epithelium. C = very few, large cells.

### Database

Relevant data, including clinical and embryonic features, as well as treatment outcomes for all PGT cycles, were extracted from electronic patient records and recorded in a database. In total, 52 continuous variables (for example, age), categoric variables (for example, stimulation protocol), or discrete variables (for example, number of embryos).

### Statistical analysis

Data were analyzed with the use of the statistical packages R (The R Foundation; http://www.r-project.org;version 3.4.3) and Empower (R) (www.empowerstates.com, X&Y solutions, inc. Boston, Massachusetts). Categorical data are represented as frequencies and percentages; variables in these measures were compared between the study groups using chi-square or Fisher’s exact tests. The ordinal categorical variable is analyzed by the Kruskal-Wallis test. Continuous data are expressed as mean ± standard deviation, compared with one-way analysis of variance (ANOVA). The study population was divided into the training and validation cohorts in a 3:1 ratio. In the model-development phase, we first perform a univariate logistic regression analysis of all variables in the training cohort. For the variables at a statistically significant level (*p* < 0.05), we carried out a variance inflation factor (VIF) test, and excluded the variables causing potential multicollinearity according to the criteria of VIF > 5. We conducted an extreme gradient boosting (XGBoost) model to analyze the contribution (gain) of each variable. We selected clinical and laboratory variables to construct a predictive model through multivariable logistic regression. We constructed the area under the receiver operator characteristics (ROC) curve (AUC). Sensitivity, specificity, positive predictive value (PPV), negative predictive value (NPV) are also presented. We also formulated nomograms for the practical application. The performance of the nomogram was quantified with respect to calibration and discrimination for external validation.

## Results

A total of 1610 embryos were biopsied, 1545 had known ploidy status (641 were euploid). The overall euploid rate was 39.8%. Baseline characteristics of the study population by training and validation cohort are shown in Table 1. We observed a significant difference between the two cohorts of uneven cleavage embryos. The embryos in the training cohort were more uneven on D3. A correlation matrix constructed is shown in Supplemental Table 1. We calculate the correlation value.

**TABLE 1:** Baseline characteristics of training and validation cohort

	Training cohort (***n*** = 1233)	Validation cohort (***n*** = 312)	***P***-value
Female age (y)	30.40 ± 4.06	30.39 ± 4.29	0.980
Male age (y)	31.89 ± 4.68	31.80 ± 4.55	0.756
Infertility duration (y)	2.36 ± 2.18	2.48 ± 2.36	0.393
Infertility type			0.933
Primary infertility	422 (34.23%)	106 (33.97%)	
Secondary infertility	811 (65.77%)	206 (66.03%)	
AFC (n)	13.70 ± 5.42	13.03 ± 5.15	0.049
Chromosomal abnormality	354 (28.71%)	78 (25.00%)	0.192
FORT (%)	87.24	83.84	0.415
FOI (%)	74.83	73.48	0.892
MII oocyte rate (%)	74.28	76.43	0.226
Cleavage rate (%)	80.03	81.19	0.193
2PN rate (%)	78.32	79.39	0.115
Blastocyst formation rate (%)	64.34	64.73	0.987
Gn dosage (IU)	2203.78 ± 750.40	2276.20 ± 833.32	0.137
Gn duration (days)	10.31 ± 1.82	10.30 ± 1.67	0.890
Euploid rate (%)	513 (41.61%)	128 (41.03%)	0.853
No. of day3 cells	8.08 ± 1.56	8.21 ± 1.66	0.188
Uneven cleavage embryos	501 (40.63%)	106 (33.97%)	0.031
Embryo fragmentation of D3			0.517
0	513 (41.61%)	141 (45.19%)	
5–10	466 (37.79%)	110 (35.26%)	
> 10	254 (20.60%)	61 (19.55%)	
Timing of embryo biopsy			0.630
D5 AM	701 (56.85%)	174 (55.77%)	
D5 PM	325 (26.36%)	90 (28.85%)	
D6	207 (16.79%)	48 (15.38%)	
Expansion degree of D5			0.554
2	7 (0.57%)	2 (0.64%)	
3	299 (24.25%)	75 (24.04%)	
4	893 (72.42%)	227 (72.76%)	
5	24 (1.95%)	3 (0.96%)	
6	10 (0.81%)	5 (1.60%)	
Inner cell mass of D5			0.064
A	9 (0.73%)	3 (0.96%)	
B	832 (67.48%)	231 (74.04%)	
C	392 (31.79%)	78 (25.00%)	
Trophectoderm grade of D5			0.930
A	6 (0.49%)	2 (0.64%)	
B	358 (29.03%)	92 (29.49%)	
C	869 (70.48%)	218 (69.87%)	

*FORT* Follicular output rate, *FOI* follicle-to-oocyte index

Arithmetic mean (95% CI)/N (%) calculated using EmpowerStats (www.empowerstats.com) and R. Kruskal-Wallis rank test for continuous variables

### Variable importance in the prediction model

The variable importance is a scaled measure to have a maximum value of 100. The predictors with variable importance of the top 20 are shown. Metaphase II (MII) oocyte rate, follicle-to-oocyte index (FOI), and follicular output rate (FORT) were the most important predictor variables in the prediction model (Supplemental Fig. 1).

### Development and validation of the prediction model

In the model-development phase, the euploid-prediction model developed according to female age, male age, parent abnormal chromosome, infertility type, infertility duration, follicular output rate (FORT), follicle-to-oocyte index (FOI), MII oocyte rate, cleavage rate, 2PN rate, blastocyst formation rate, AFC, the cell number of D3, uneven cleavage embryos, embryo fragmentation of D3, the timing of embryo biopsy, expansion degree of D5, inner cell mass of D5, trophectoderm grade of D5, gonadotropin (Gn) dosage, and Gn duration showed good discriminatory power with AUC of 0.859 (95% CI, 0.834–0.872), the sensitivity of 90.31%, specificity of 57.84%, accuracy 89.59%, positive predictive value (PPV) of 75.13% and negative predictive value (NPV) of 80.92% (Table 2).

**TABLE 2:** Accuracy of the prediction model in the training and validation cohort

	Training cohort (***n =*** 1233)	Validation cohort (***n =*** 312)
AUC	0.859 (0.834,0.872)	0.852 (0.831,0.879)
Sensitivity, %	90.31	92.15
Specificity, %	57.84	50.33
Accuracy, %	89.59	89.24
Positive predictive value, %	75.13	73.21
Negative predictive value, %	80.82	84.05

*AUC* Area under the curve

In the model validation phase, we observed good discriminatory powers with the AUCs of 0.852 (95% CI, 0.831–0.879), the sensitivity of 92.15%, specificity of 50.33%, accuracy 89.24%, positive predictive value (PPV) of 73.21% and negative predictive value (NPV) of 84.05%.

**Fig. 1:**
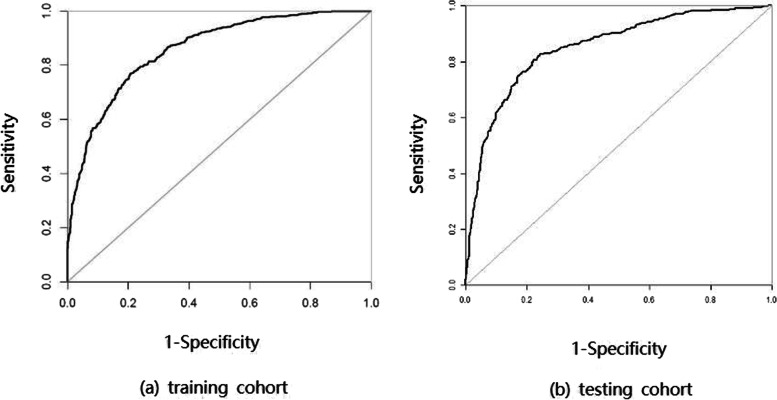
ROC curve of the nomogram to predict euploid. (a) ROC curve in training cohort. (b) ROC curve in validation cohort

**Fig. 2:**
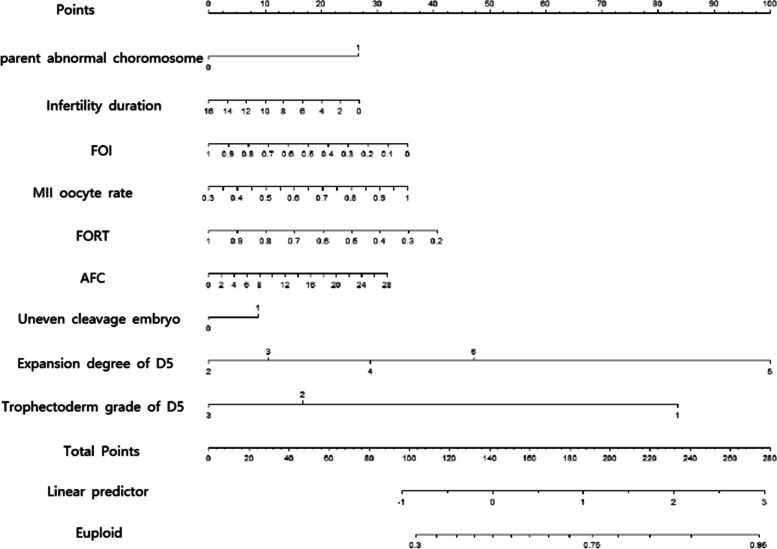
Nomogram to predict euploid in patients undergoing PGT-A

The ROC of the prediction model in the training and validation cohort was plotted in Fig. 1. The nomogram of the model was drawn to provide quantitative and convenient tools in predicting euploid embryos by clinical and laboratory characteristics (Fig. 2). The nomogram was subjected to bootstrapping with 500 resamples. Find the score for each variable for an individual embryo on the uppermost rule (“Points”). Add all scores together and find the sum of the scores on the “Total points” rule.

### Calibration curves

The calibration curve of the full model was plotted to evaluate the consistency between the predicted probability of euploid and actual results are presented in Supplemental Fig. 2. The bias curve is close to the ideal line in the figure, and good agreement can be observed between the prediction and observation.

## Discussion

This training cohort included 1233 embryos with the euploid rate of 41.61%. We established a euploid-prediction model to predict euploid embryos for patients undergoing IVF. Most studies focused on clinical outcomes of the women, however, we established a euploid prediction model for non-invasive embryo selection with high AUC. The model exhibited relatively good discriminatory power and the verification was also satisfactory. Furthermore, our model can be used to patient-specifically rank blastocysts on euploid. The training and test sets were randomly selected multiple times to reduce the impact of discrepancies.

We found that MII oocyte rate, FOI, and FORT were the most important predictor variables in predicting euploid. Previous research suggested the MII oocytes rate from large follicles is significantly higher than from small follicles [13]. MII oocyte rate rather than the number of oocytes retrieved is a more accurate predictor of implantation and clinical pregnancy in IVF cycles [14]. FOI is defined as the ratio between the number of oocytes collected at the oocyte retrieval and AFC at the beginning of ovarian stimulation. FOI is proposed as a novel parameter to assess ovarian response [15]. FOI could reflect the dynamic nature of follicular growth and could represent a tool to determine whether the ovarian reserve was adequately exploited during ovarian stimulation. FORT is defined as the ratio between pre-ovulatory follicle count on hCG and AFC. A previous study showed FORT and FOI were different in different ovarian responders [16]. FORT can effectively predict pregnancy outcome, and not be affected by factors such as age and BMI [17].

A previous study developed a prediction model for aneuploidy by a 12 – gene transcriptomic signature using data from a small number of embryos (*n* = 48), however, it is not intended for clinical use, but to identify cellular pathways and related molecules indicative of the embryo ploidy status [18]. Previous research to establish predictive models were problematic due to the limited number of involved characteristics [19, 20]. Kaufmann et al. reported a neural network predicting model for patients undergoing IVF treatment, however, the overall accuracy was low, with only 59% [21]. Uyar et al. reported a Support Vector Machine (SVM) method for the classification of embryos according to implantation potentials, with an AUC of 0.712 ± 0.032 when 12 features were included [22].

XGBoost is a newly developed algorithm, with higher calculating speed and accuracy. Many clinical prediction models have been established using XGBoost and were proved to be better than traditional statistical approaches [23–25]. The euploid-prediction model could help doctors to identify ploidy-status with clinical and laboratory characteristics. We observed relatively high sensitivity and NPV, which means a lower likelihood of missing euploid; a relatively high PPV, which means a lower likelihood of misjudging embryos with actually euploid. This model can be used as a reference for embryo selection in patients undergoing IVF.

This study has several advantages. First, based on the clinical and laboratory characteristics of embryos, more factors were used as predictors to construct the prediction model for ploidy status. Second, we use all embryos that underwent PGT-A instead of transferred embryos, since not all tested embryos could be transferred into the uterus and have pregnancy outcomes. Finally, our prediction model provides a relatively accurate, convenient, and noninvasive method for embryo screening, applicable to patients who refuse PGT-A. Therefore, this euploid prediction model is expected to solve the problem of embryo selection in clinical work.

There are some limitations to our study. First, this was a retrospective study, so inherent biases and variations were inevitable. Therefore, further verification is needed to ensure the better robustness of the model. Second, models established by different methods should be compared and combined to develop a model with optimal prediction performance. Thirdly, this is a single-center study, limiting the generalizability of the study, prospective multi-center studies are needed to further verify the current findings. Finally, our prediction model had higher sensitivity but substantially lower specificity. These results suggest that this euploid prediction model despite having good performance may not be useful in accurately determining the aneuploidy (due to low specificity). However, a recent study has demonstrated a comparable cumulative live birth rate in women undergoing conventional IVF and PGT-A, indicating PGT-A may potentially encourage the waste of healthy embryos [26]. As the goal of PGT-A is to select the best quality embryos for transfer, the high sensitivity of selecting euploid will decrease embryo waste.

## Conclusions

We built a euploid prediction model based on clinical and laboratory characteristics of the embryos to predict ploidy status, which exhibited relatively satisfactory discriminatory powers in verification. Our model may help clinicians better select embryos with noninvasive methods.

## Supplementary Information

**Additional file 1: Supplemental Fig 1.** Importance of the predictor variables, scaled to a maximum of 100

**Additional file 2: Supplemental Fig 2.** Calibration curve of the full model

**Additional file 3: Supplemental Table 1.** Correlation matrix of the features

## Data Availability

The datasets used and/or analyzed during the current study are available from the corresponding author on reasonable request.

## Abbreviations

PGT-A: preimplantation genetic testing for aneuploidies
ROC: receiver operating characteristic
