# Supplementary figures and images for: Nomogram based on clinical and laboratory characteristics of euploid embryos using the data in PGT-A: a euploid-prediction model
Source: BMC Pregnancy Childbirth. 2022 Mar 17;22:218. doi: 10.1186/s12884-022-04569-3 (PMC8932287; doi:10.1186/s12884-022-04569-3)

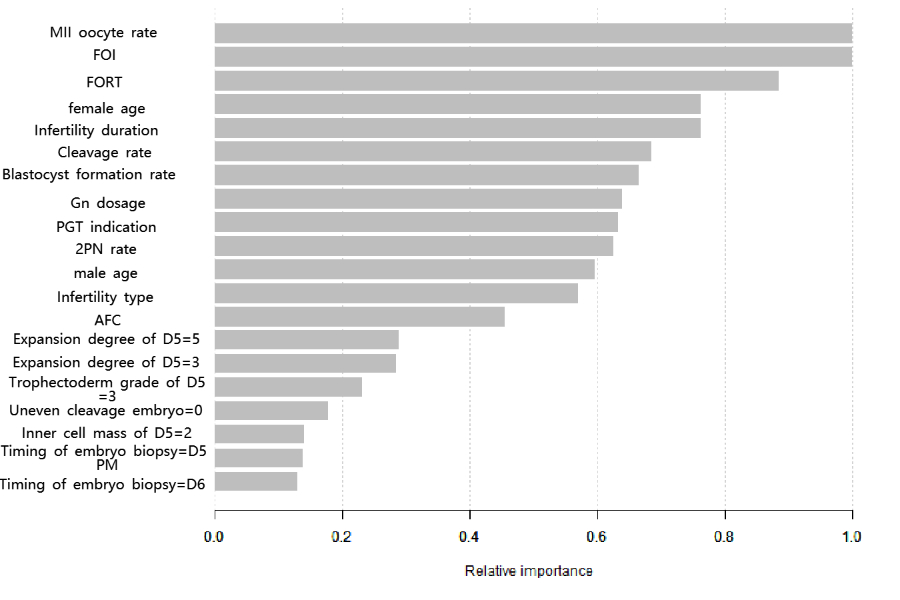

Supplement: Supplementary file 1 — Additional file 1: Supplemental Fig 1. Importance of the predictor variables, scaled to a maximum of 100 [file 12884_2022_4569_MOESM1_ESM.tif]

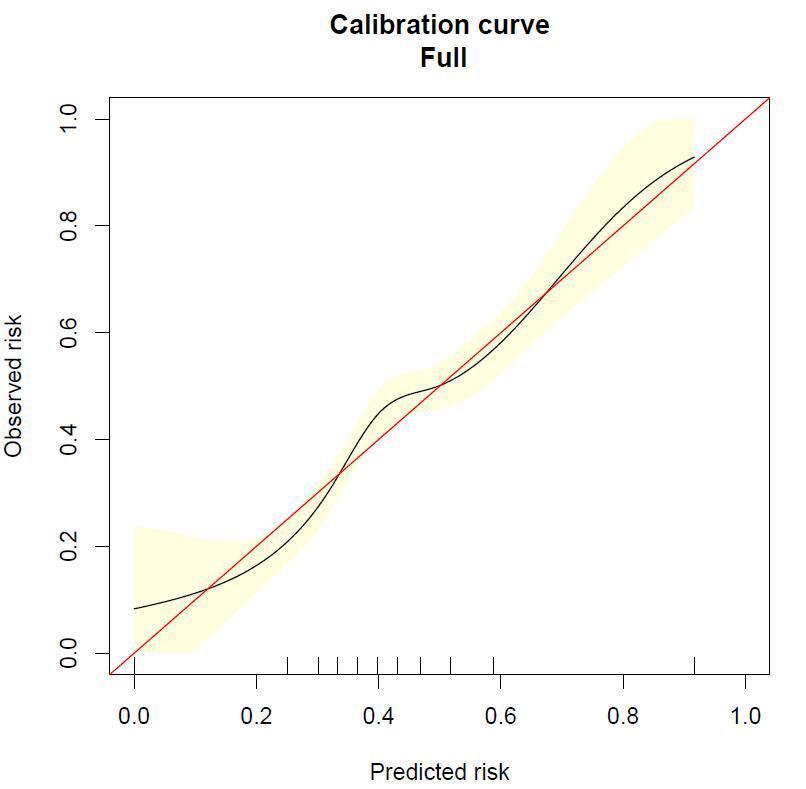

Supplement: Supplementary file 2 — Additional file 2: Supplemental Fig 2. Calibration curve of the full model [file 12884_2022_4569_MOESM2_ESM.tif]
