# Supplementary material for: Nomogram based on clinical and laboratory characteristics of euploid embryos using the data in PGT-A: a euploid-prediction model
Source: BMC Pregnancy Childbirth. 2022 Mar 17;22:218. doi: 10.1186/s12884-022-04569-3 (PMC8932287; doi:10.1186/s12884-022-04569-3)
Supplement: Supplementary file 3 — Additional file 3: Supplemental Table 1. Correlation matrix of the features [file 12884_2022_4569_MOESM3_ESM.docx]

**Supplemental TABLE 1 Correlation matrix of the features.**

|  | Female age | Male age | FORT | FOI |
| --- | --- | --- | --- | --- |
| Female age |  |  |  |  |
| Male age | 0.21 |  |  |  |
| FORT | -0.19 | 0.08 |  |  |
| FOI | -0.11 | -0.17 | 0.39 |  |
